# Molecular Assessment of Proadipogenic Effects for Common-Use Contraceptives and Their Mixtures

**DOI:** 10.1210/endocr/bqae050

**Published:** 2024-04-22

**Authors:** Yu-Ting Tiffany Chiang, Christopher D Kassotis

**Affiliations:** Institute of Environmental Health Sciences and Department of Pharmacology, Wayne State University, Detroit, MI 48202, USA; Institute of Environmental Health Sciences and Department of Pharmacology, Wayne State University, Detroit, MI 48202, USA

**Keywords:** birth control, contraceptives, metabolism disrupting chemicals, adipogenesis, mixtures, obesogen

## Abstract

Hormonal contraceptives are widely prescribed due to their effectiveness and convenience and have become an integral part of family planning strategies worldwide. In the United States, approximately 65% of reproductive-aged women are estimated to be using contraceptive options, with approximately 33% using one or a combination of hormonal contraceptives. While these methods have undeniably contributed to improved reproductive health, recent studies have raised concerns regarding their potential effect on metabolic health. Despite widespread anecdotal reports, epidemiological research has been mixed as to whether hormonal contraceptives contribute to metabolic health effects. As such, the goals of this study were to assess the adipogenic activity of common hormonal contraceptive chemicals and their mixtures. Five different models of adipogenesis were used to provide a rigorous assessment of metabolism-disrupting effects. Interestingly, every individual contraceptive (both estrogens and progestins) and each mixture promoted significant adipogenesis (eg, triglyceride accumulation and/or preadipocyte proliferation). These effects appeared to be mediated in part through estrogen receptor signaling, particularly for the contraceptive mixtures, as cotreatment with fulvestrant acted to inhibit contraceptive-mediated proadipogenic effects on triglyceride accumulation. In conclusion, this research provides valuable insights into the complex interactions between hormonal contraceptives and adipocyte development. The results suggest that both progestins and estrogens within these contraceptives can influence adipogenesis, and the specific effects may vary based on the receptor disruption profiles. Further research is warranted to establish translation of these findings to in vivo models and to further assess causal mechanisms underlying these effects.

Hormonal contraceptives, such as combined oral contraceptives, progestin-only pills, and contraceptive patches, as well as intrauterine devices (IUDs), have been widely prescribed due to their effectiveness and convenience and have become an integral part of family planning strategies worldwide. Additionally, emergency contraception, often containing high doses of hormones, offers an essential option for preventing unintended pregnancies. In the United States, approximately 65% of reproductive-aged women are estimated to be using contraceptive options, with approximately 33% using one or a combination of hormonal contraceptives (1). While these methods have undeniably contributed to improved reproductive health, recent studies have raised concerns regarding their potential effect on metabolic health (2-6). Weight gain remains the top reported reason for discontinuation of hormonal contraceptives, with approximately 33% of hormonal contraceptive users reporting that they discontinued use due to weight gain concerns. Despite widespread anecdotal reports, epidemiological research has been mixed as to whether hormonal contraceptives contribute to metabolic health effects (7, 8).

Surprisingly little research exists that has examined the effects of hormonal contraceptive chemicals on metabolic health outcomes, particularly when taken at low concentrations over long periods of time. Interestingly, several Cochrane reviews have evaluated the effect of contraceptive use on weight gain. The first (initially published in 2003 and revised most recently in 2014) broadly examined combination contraceptive pills and patches’ effect on weight, finding that most combined contraceptives showed either minimal or no differences in weight (9). The second (initially published in 2010 and revised most recently in 2016) focused on progestin-only contraceptives, reporting low-quality/limited evidence for effects on body weight or composition, though it noted that a number of included studies (mostly downrated for lack of randomization or high rate of dropout) did report modest weight gain in participants (10). While these previous assessments have been relatively inconclusive, they have failed to properly account for molecular differences between specific hormonal contraceptives. Other studies, assessing more specific contraceptives, or more discrete health outcomes, reported increased weight gain, regain after weight loss, and/or disrupted bone density/mass (2-6).

Despite a paucity of robust data in humans, there is ample evidence available in animal studies, where estrogens and/or other hormones have been used for fattening livestock for more than 80 years (11-13). There is also a wealth of evidence on the metabolism-disrupting effects of diethylstilbestrol, a model estrogen, in vitro, in vivo, and in human epidemiological studies (14-16). While the use of estrogens in fattening chickens was restricted by the US Food and Drug Administration in the 1950s, it was common practice before then and resulted in greatly enhanced adipose in the animals (12, 17, 18). There is also a considerable history of diethylstilbestrol use for fattening cattle, with observations of growth stimulation observed that resulted in US Food and Drug Administration approval in 1954 and withdrawal in 1972, following epidemiological findings and reports of diethylstilbestrol residues in cattle (19, 20). Interestingly, diethylstilbestrol was replaced by a variety of growth-promoting chemicals in cattle, including androgens, estrogens, and progestins, among others (21, 22), supporting the utility of estrogens and progestins in promoting weight. Outside agriculture, there is a wealth of other experimental evidence for contraceptive and other estrogens promoting weight gain and altering metabolic profile in fish (23) and other species (24-26). A variety of progestin contraceptives have also been demonstrated to increase weight, adiposity, and/or disrupt the metabolic profile in rodents and other laboratory animals, including medroxyprogesterone acetate (27, 28). Importantly, while some of the current hormonal contraceptives have been reported in preclinical studies to have metabolic effects, others have reported net positive effects and no reported effects on weight gain or adiposity.

Even fewer data are available on the effects of hormonal contraceptives on children exposed during gestation. Approximately 9% of hormonal contraceptive users become pregnant inadvertently, due to mistakes in or failure of dosing, drug interactions, or other factors; these women often inadvertently expose their fetus to hormonal contraceptives prior to understanding their conception status. Limited research has described potential associations between early pregnancy use of specific hormonal contraceptives and offspring overweight or obesity by age 3 years (29), demonstrating some evidence for lasting effects on offspring metabolic health outcomes. Another study reported increased birthweight and placental weights, seemingly associated with higher serum progesterone and estradiol concentrations, for women with prior contraceptive use (30). Some research has demonstrated increased risk for developmental defects (eg, increase in congenital urinary tract abnormalities for oral contraception use after conception (31)), but other studies (32) as well as a meta-analysis failed to find any significant associations (33). There have been some reports of associations between specific hormonal contraceptive use around the time of conception (eg, generally for specific progestins) and increased risk of preterm birth (34) as well as increased low and very low birth weights (35). Some evidence also suggests increased risk for epilepsy in children born to mothers who had prescriptions for recent hormonal contraception near the time of conception, which was strongest for progestin-only products and combined products (36). Other studies have reported positive relationships between progestin-only contraceptive use in the year prior to pregnancy and increased offspring wheezing at age 6 to 8 months (37), as well as increased childhood attention-deficit/hyperactivity disorder (ADHD) incidence (highest with greatest recency to time of conception and for progestin products, specifically) (38). An increased risk for specific cancers (eg, nonlymphoid leukemia) (39) has also been observed, along with research suggesting poorer prognosis associated with these cancers and higher mortality risk (40). Many of these outcomes have been demonstrated only in single cohorts, often with other studies reporting negative outcomes; thus, these preliminary developmental effects should be interpreted with caution (though they warrant further research).

The goals of this study were to assess the adipogenic activity of common hormonal contraceptive chemicals and their mixtures, as well as to determine their presumed causal mechanism of action by comprehensive evaluation of bioactivities using coexposure experiments. Five different models of adipogenesis were used to provide a rigorous assessment of metabolism-disrupting effects. There is an expanding body of work identifying diverse environmental contaminants as metabolism disruptors able to directly modulate metabolic health end points in vitro and/or in vivo (41-45). With metabolic disorders, such as obesity, vastly increasing in incidence (obesity affecting >42% of US adults, >70% obese and overweight (46)), it is imperative to characterize how metabolism-disrupting environmental contaminants may be exacerbating this pandemic. In particular, a diverse group of therapeutic chemicals are prescribed widely and used over long-periods of time by reproductive-aged women (among others, for nonreproductive therapeutic use for other reported health concerns).

## Materials and Methods

### Chemicals

Chemicals were purchased as follows: rosiglitazone (peroxisome proliferator activated receptor gamma, PPARγ, agonist; Sigma catalog No. R2408), T0070907 (PPARγ antagonist; catalog No. T8703), and contraceptive chemicals as presented in Table 1. Stock solutions were prepared in dimethyl sulfoxide (DMSO; Sigma catalog No. D2650), and all stock and working solution vials were stored at −20 °C between uses. Contraceptives in common use were determined via literature search. Mixtures of contraceptives that are marketed in products as a combination were composed of equimolar concentrations. Throughout the manuscript, a 1-μM mixture concentration will be used to denote 1 μM of each of the component chemicals.

**Table 1.** Contraceptives and control chemicals

Contraceptive chemical	Acronym	Formulation	Commercial products	Source	Catalog No.
Estrogens					
17α-Ethinyl-estradiol	EE2	Oral; combination with various progestogens	Ortho-Tricyclen, Yasmin, Yaz, Tri-Sprintec, etc	Sigma	E4876-1G
Estetrol	E4	Oral; combination with drospirenone	Nextstellis	Sigma	SML 1523-5mg
Progestins					
Segesterone acetate (Nesterone)	NES	Vaginal ring in combination with EE2	Nesterone, Elcometrine, and Annovera	Sigma	SML0550-10MG
Etonogestrel	ETON	Implant as single chemical; vaginal ring in combination with EE2	Nexplanon, Implanon (single); NuvaRing, Circlet (combination with EE2)	Sigma	SML0356-25MG
Drospirenone	DROS	Oral; both single chemical and combinations with E2, EE2, or E4	Slynd (single); Yasmin, Yaz (combination with EE2); Nextstellis (combination with E4)	Sigma	SML0147-50MG
Ulipristal acetate	UL	Oral; single chemical	Ella, EllaOne, Esmya	Sigma	SML1469-10MG
Levonorgestrel	LEVO	IUD; single chemical	Mirena, Skyla, Liletta, Kyleena	Sigma	PHR1850- 500MG
Norelgestromin	NORL	Dermal patch; combination with EE2	Evra, Ortho Evra, Xulane	Sigma	1468454-200MG
Norgestimate	NORG	Oral; combination with EE2	Ortho Tri-Cyclen, Previfem, Cilest	Sigma	PHR2119-500MG
Medroxyprogesterone acetate	MPA	Injection	Provera, Depo-Provera, Depo-SubQ Provera 104, Curretab, Cycrin, Farlutal, Gestapuran, Perlutex, Veramix	Sigma	M1629-1G
Norethindrone	NORT	Oral; single chemical	Micronor, Ortho Micronor, Nor-Q-D	Sigma	PHR1714- 1G
Norethisterone acetate	NORA	Oral; single chemical or in combination with EE2	Primolut-Nor, Aygestin, Gestakadin, Milligynon, Monogest, Norlutate, Primolut N, SH-420, Sovel, Styptin CR	Sigma	46527-100MG
Control chemicals					
17β-estradiol	E2			Sigma	E8875-250MG
Progesterone	P4			Sigma	P0130-25G

Chemical identification, ordering information, and basic physicochemical properties for each of the contraceptives and control chemicals examined in this study.

Abbreviation: IUD, intrauterine device.

### Reporter Gene Activity Bioassays

HEK-293T/17 human embryonic kidney cells (ATCC No. CRL-11268, lot No. 70022180) were maintained in growth media (Dulbecco’s modified Eagle’s medium–High Glucose [DMEM-HG], Gibco No. 11995, with 10% fetal bovine serum, Sigma F2442-500ML, and 1% penicillin and streptomycin, Gibco 15140). Ishikawa human endometrial adenocarcinoma cells (Sigma No. 99040201, lot No. 17C011) were maintained in growth media (MEM, Gibco 11090, with 5% newborn calf serum, Fisher 16010159, 1% glutamax, Gibco 35050, 1% nonessential amino acids, Gibco 11140, and 1% penicillin and streptomycin). Cells were maintained in a subconfluent state according to standard protocols. To prepare for transfection, near confluent flasks were switched to white medium (same base media without phenol red, and with charcoal-stripped fetal bovine serum) at least 2 days prior. After 2 days, flasks were treated with a combination of Lipofectamine LTX & Plus reagent (Invitrogen catalog No. 15338-100) and plasmids in Opti-MEM (Gibco 11058), then recovered overnight in assay media prepared without phenol red. Plasmids consisted of human hormone receptor constructs: peroxisome proliferator activated receptor gamma (PPARγ: pcDNA-PPARγ1), thyroid hormone receptor beta (TRβ: hTRβ1-pSG5), glucocorticoid receptor (GR: pRST7-GR), retinoid X receptor alpha (RXRα: pcDNA-RXRα), progesterone receptor B (PR B: pcDNA-PRB), and estrogen receptor alpha (ERα: N/A—not used). Reporter gene plasmids were used as follows: PPARγ: DR1-luciferase, TRβ: pGL4-TK-2X-TADR4, GR: MMTV-luciferase, RXRα: DR1-luciferase, PR B: MMTV-luciferase, and ERα: 3xERE-TK-luciferase. A constitutively-active CMV-β-Gal normalization plasmid (all plasmids were generous gifts of the Donald McDonnell Lab) was used for normalization of reporter gene bioactivity to putative cytotoxicity. PPARγ, RXRα, and TRβ plasmids were transfected into HEK293 cells; and GR, ER, and PR into Ishikawa cells. The following morning, transfected cells were seeded at approximately 60 000 cells per well into 96-well tissue culture plates and allowed to settle. Settled cells were then induced with dose responses of positive and/or negative controls and test chemicals using a 0.1% DMSO vehicle. Cells were treated for approximately 20 hours and then lysed for luciferase and β-galactosidase assays.

To calculate effects, raw luminescence values were converted to fold inductions relative to the solvent control responses (0.1% DMSO) and were then used to calculate percentage bioactivities relative to positive control agonists and/or antagonists. For agonist bioassays, chemical values were compared to the maximal positive control-induced responses to determine percentage activity. For antagonist bioassays, percentage activity was calculated as percentage enhancement or suppression relative to the half maximal positive control-induced responses (as determined in agonist bioassays). Significant reduction in constitutively active β-galactosidase promoter activity (>15%) was used as an indirect marker of toxicity and receptor bioactivities were reported only in the absence of presumed toxicity.

### Adipogenesis Cell Care and Bioassays

3T3-L1 cells (Zenbio catalog No. SP-L1-F, lot No. 3T3062104) were maintained in preadipocyte media (DMEM-HG; Gibco No. 11995, with 10% bovine calf serum and 1% penicillin/streptomycin; Gibco No. 15140) at a subconfluent state as described previously (47-51) and used between passages 8 and 12. 3T3-L1 cells were seeded at approximately 30 000 cells per well into 96-well tissue culture plates, grown to confluency, and then allowed 48 hours for growth arrest and clonal expansion. Differentiation was induced by replacing media with test chemicals and/or controls using a DMSO vehicle (at 0.1%) in differentiation media (DMEM-HG with 10% fetal bovine serum, 1% penicillin/streptomycin, 1.0 μg/mL human insulin, and 0.5 mM 3-isobutyl-1-methylxanthine, IBMX). After 48 hours of differentiation induction, media were replaced with fresh dilutions of test chemicals and/or control chemicals in adipocyte maintenance media (differentiation media without IBMX), and these media were refreshed every 2 to 3 days until assay, 10 days after induction.

All 4 lots of human mesenchymal stem cells (hMSCs; Table 2) were induced to differentiate according to a unified protocol (52-54). Briefly, cells were seeded in basal media (DMEM/Nutrient Mixture F-12, DMEM/F-12; Gibco No. 11320, with 10% fetal bovine serum and 1% penicillin/streptomycin) into 96-well plates at approximately 10 000 cells per well. After settling for at least 1 day, media were replaced with test chemicals diluted in differentiation media as described earlier (Zenbio catalog No. DM-2-500). Differentiation media were left undisturbed for 3 days and then removed and replaced with fresh test chemical dilutions in adipocyte maintenance media (Zenbio catalog No. AM-1); this was refreshed every 3 to 5 days for a further 18 days until assay at day 21.

**Table 2.** Description of human mesenchymal stem cells

Cell code	Cell provider	Catalog No.	Lot No.	Sex	Age	Race
Female 1	Zenbio Inc	HBMMSC-F	HBMMSC071819A	Female	35 y	White
Female 2	ScienCell	7500	31886	Female	20 wk	Not available
Male 1	Lonza Inc	PT-2501	19TL155677	Male	31 y	Black
Male 2	ScienCell	7500	30579	Male	20 wk	Not available

Basic demographic and descriptive data for human mesenchymal stem cell lots used in this study.

Plates were processed for measurements of triglyceride accumulation and DNA content as described in detail previously (47-51). Briefly, media were removed from all wells and cells were rinsed with Dulbecco's phosphate-buffered saline (DPBS). Dye mixture was then added (200 μL per well; 19 mL DPBS; 20 drops NucBlue reagent, Thermo catalog No. R37605; and 500 μL Nile Red solution, 40 μg/mL in acetone, Sigma catalog No. 72485-100MG). Plates were incubated, protected from light for 30 to 40 minutes at room temperature, then fluorescence was measured using a Molecular Devices SpectraMax iD5 microplate spectrofluorimeter (485 nm/572 nm excitation/emission for Nile Red and 360/460 for NucBlue). Triglyceride accumulation was calculated as fold induction relative to the intra-assay–differentiated vehicle control response (0.1% DMSO) and then as percentage activity relative to the maximum rosiglitazone-induced response. DNA content was calculated as percentage change (positive values, preadipocyte proliferation; negative values, cytotoxicity) from the differentiated vehicle control responses, and then was used to normalize triglyceride accumulation. Three biological replicates (separate assays and cell passages), each including 4 technical replicates (intraplate replicates), were performed for all testing.

### Statistical Analysis

Cell data are presented as means ± SEM from 4 technical replicates of 3 independent biological replicates. Kruskal-Wallis with Dunn's multiple comparisons test was performed to determine significant differences across concentrations and relative to DMSO controls (*P* < .05 considered statistically significant). Statistical comparisons were made using GraphPad Prism 10.0.

## Results

### Steroidal Receptor Bioactivities

**Figure 1.**
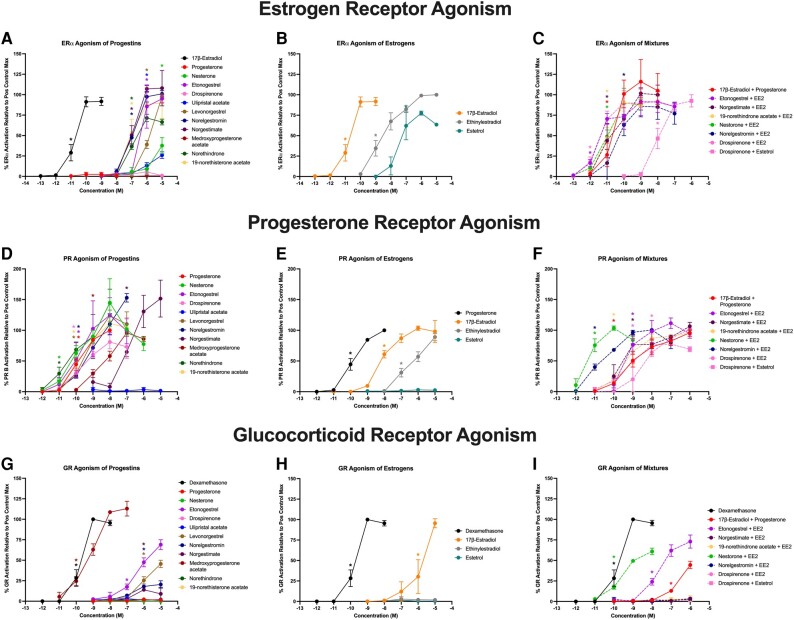
Steroid hormone receptor bioactivities for contraceptives and their mixtures. Each current-market estrogen and progestin as well as their mixtures were tested in transiently transfected reporter gene assays in human cell lines as described in “Materials and Methods” and assessed for percentage agonism on each receptor relative to the positive control agonist–induced maximal response. Estrogen receptor alpha agonism (percentage activity relative to 17β-estradiol–induced maximum) for each A, progestin; B, estrogen; and C, each combined mixture. Progesterone receptor B agonism (percentage activity relative to progesterone-induced maximum) for each D, progestin; E, estrogen; and F, each combined mixture. Glucocorticoid receptor agonism (percentage activity relative to dexamethasone-induced maximum) for each G, progestin; H, estrogen; and I, each combined mixture. Data are presented as mean ± SEM from 3 independent experiments. *Indicates lowest concentration with significant increase over vehicle control response; *P* less than .05, as per Kruskal-Wallis in GraphPad Prism 10.

A set of common contraceptive chemicals and their mixtures were tested in human receptor reporter gene assays to measure agonism of PPARγ, TRβ, GR, RXRα, PR B, and ERα and antagonism of TRβ. Given the presumed mechanisms of action for the contraceptive chemicals, we first evaluated agonism for ERα, PR B, and GR. Of the 11 progestins evaluated, 8 of these demonstrated significant activation of ERα (progesterone, medroxyprogesterone acetate, and drospirenone had no significant activity for ERα); these compounds exhibited a range of bioactivities ranging from 26% to 108% relative to the maximal estradiol-induced response (Fig. 1A). Each of the 3 tested estrogens demonstrated robust activation of ERα, with activation ranging from 78% to 100% relative to the maximal response (Fig. 1B). For the 8 examined mixtures, each of them significantly activated ERα, with the drospirenone + estetrol (E4) mixture having 100- to 1000-fold lower potency than the other mixtures, and activities ranging from 85% to 116% (Fig. 1C). For activation of the PR B, 10 of the 11 progestins significantly activated this receptor, with bioactivities ranging from 81% to 153% relative to the progesterone-induced maximum (Fig. 1D). Ulipristal acetate was the only presumed progestin that did not activate PR (reported antiprogestin mechanism of action). Two of the 3 estrogens (with the exception of E4) also significantly activated PR, with activities ranging from 89% to 104%, and orders of magnitude lower potencies than progesterone (Fig. 1E). Each of the 8 examined mixtures significantly activated PR, with bioactivities ranging from 77% to 112% and potencies spanning 10 pM (EC50; nestorone + ethinylestradiol [EE2]) to 10 nM (drospirenone + EE2; Fig. 1F). At higher concentrations, many PR ligands can cross over and activate the GR directly, so this was also examined for each of the contraceptives and mixtures. Five of the 11 progestins significantly activated GR, with medroxyprogesterone acetate inducing an equivalent response to the positive control, dexamethasone, and the 4 other active progestins inducing responses that were 1000-fold less potent and ranging from 14% to 69% efficacy (Fig. 1G). Only estradiol (E2) activated GR at 96% relative to the maximal dexamethasone-induced response, though with a 10 000-fold less potent response (Fig. 1H). Three of the 8 mixtures significantly induced GR, with nestorone + EE2 inducing the most potent response but only at 61% efficacy, etonogestrel + EE2 inducing a 73% response at 100-fold lower potency, and E2 + progesterone inducing 44% response and 1000-fold lower potency (Fig. 1I).

### Metabolism-relevant Receptor Bioactivities

**Figure 2.**
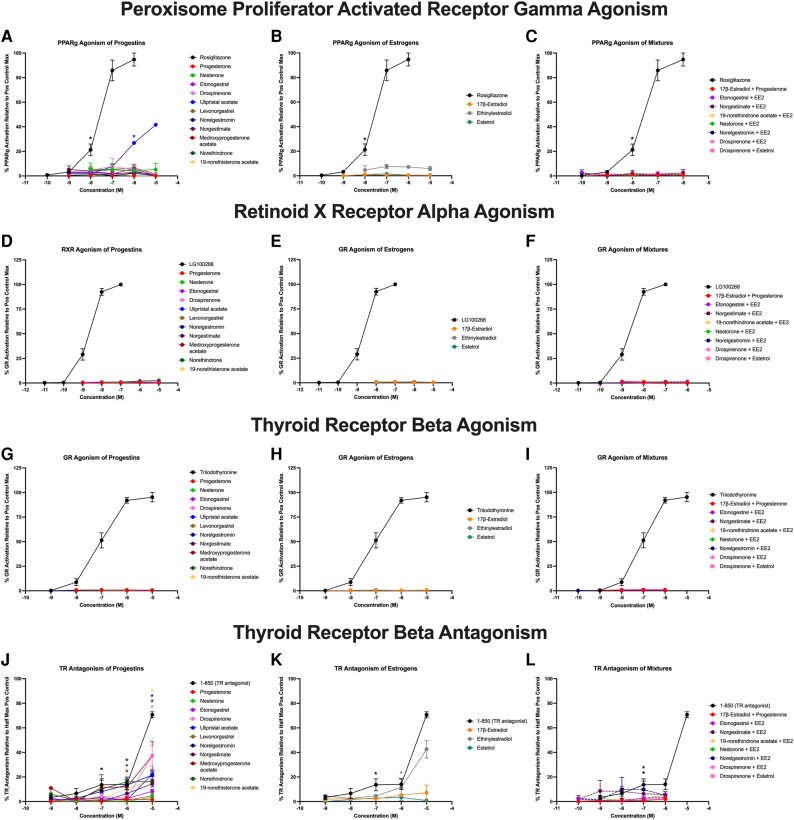
Nuclear hormone receptor bioactivities for contraceptives and their mixtures. Each current-market estrogen and progestin as well as their mixtures were tested in transiently transfected reporter gene assays in human cell lines as described in “Materials and Methods” and assessed for percentage agonism or antagonism (as noted) on each receptor relative to the positive control agonist–induced maximal response. Peroxisome proliferator activated receptor gamma agonism (percentage activity relative to rosiglitazone-induced maximum) for each A, progestin; B, estrogen; and C, each combined mixture. Retinoid X receptor alpha agonism (percentage activity relative to LG100268-induced maximum) for each D, progestin; E, estrogen; and F, each combined mixture. Thyroid receptor beta agonism (percentage activity relative to triiodothyronine-induced maximum) for each G, progestin; H, estrogen; and I, each combined mixture. Thyroid receptor beta antagonism (percentage inhibition of half maximal triiodothyronine-induced response) for each J, progestin; K, estrogen; and L, each combined mixture. Data are presented as mean ± SEM from 3 independent experiments. *Indicates lowest concentration with significant increase over vehicle control response; *P* less than .05, as per Kruskal-Wallis in GraphPad Prism 10.

We next evaluated each of the chemicals and mixtures for agonism of PPARγ and RXRα and both agonism and antagonism of TRβ. Only one progestin, ulipristal acetate, induced significant activation of PPARγ, with efficacy of 42% relative to the maximal rosiglitazone-induced response and EC50 of approximately 600 nM (Fig. 2A). No other progestins, estrogens, or mixtures activated PPARγ (Fig. 2A-C). None of the progestins, estrogens, or mixtures acted as agonists for either RXRα (Fig. 2D-F) or TRβ (Fig. 2G-I). However, 7 of the 11 progestins significantly antagonized TRβ (Fig. 2J), with drospirenone and medroxyprogesterone acetate inducing the most robust effects (∼37% inhibition of the half-maximal triiodothyronine response), and ulipristal acetate, norelgestromin, norethindrone, norethisterone acetate, and norgestimate exhibiting antagonism ranging from 14% to 22%. One of the estrogens antagonized TRβ, with EE2 exhibiting 43% antagonism and the others exhibiting no significant activity (Fig. 2K). Only 1 of the 8 mixtures significantly antagonized TRβ, with norelgestromin + EE2 inducing approximately 14% antagonism (Fig. 2L).

### Adipogenic Activity Determinations

**Figure 3.**
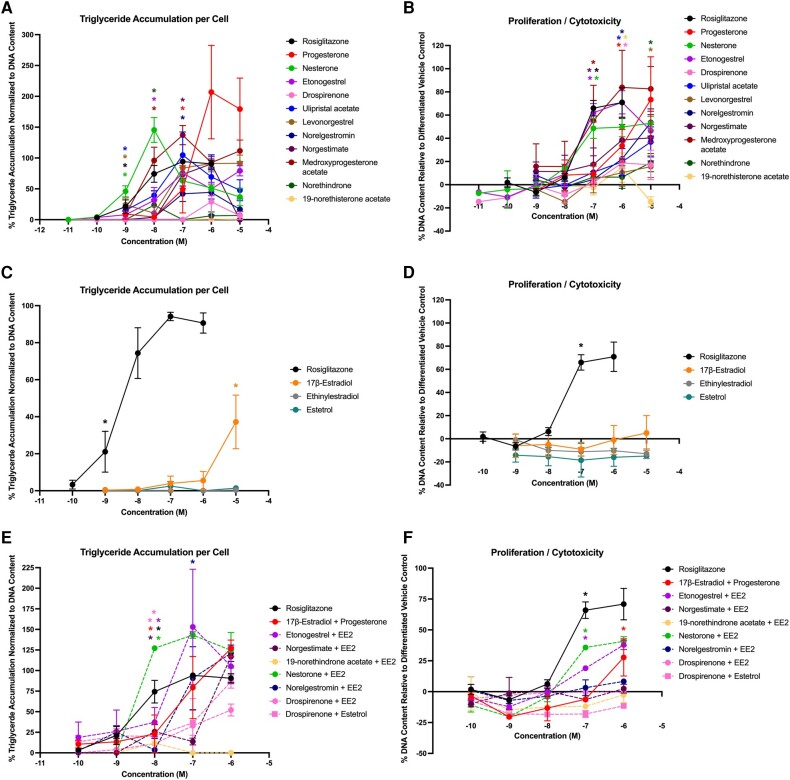
Hormonal contraceptives and mixtures induce adipogenic activity in 3T3-L1 cells. Zenbio 3T3-L1 cells were differentiated as described in “Materials and Methods” and assessed for adipocyte differentiation (Nile Red staining of lipid accumulation) and cell proliferation (Hoechst staining) after 10 days of differentiation while exposed to various contraceptives or their mixtures at a range of concentrations. Percentage normalized triglyceride accumulation per well relative to maximal response for rosiglitazone (normalized to DNA content; A, C, E). Increase (cell proliferation) or decrease (potential cytotoxicity) in DNA content relative to vehicle control (B, D, F). Contraceptive progestins (A, B), estrogens (C, D), and combined contraceptive mixtures (E, F). Data are presented as mean ± SEM from 3 independent experiments. *Indicates lowest concentration with significant increase over vehicle control response; *P* less than .05, as per Kruskal-Wallis in GraphPad Prism 10.

Given the apparent bioactivities on key metabolic health pathways, we next examined the adipogenic effects of these chemicals and their mixtures in the murine 3T3-L1 preadipocyte model. Interestingly, 9 of the 11 progestins exhibited significant triglyceride accumulation relative to the rosiglitazone-induced maximal response (Fig. 3A; Table 3). Drospirenone and 19-norethisterone acetate exhibited no significant activity on triglyceride accumulation, whereas progesterone and 8 of the other progestins exhibited activity ranging from 24% to 207%. Each of the 11 progestins exhibited significant effects on preadipocyte proliferation, with effects ranging from 15% to 84% increased DNA content relative to the differentiated solvent control (Fig. 3B). While 17β-estradiol induced significant triglyceride accumulation (37% relative to the rosiglitazone-induced maximum), neither of the other estrogens induced any effect on triglyceride accumulation (Fig. 3C). None of the estrogens exhibited any significant effect on preadipocyte proliferation (Fig. 3D). For the mixtures, 7 of the 8 mixtures induced significant triglyceride accumulation ranging from 52% to 153% (Fig. 3E) and 3 of the 8 induced significant effects on preadipocyte proliferation ranging from 19% to 41% (Fig. 3F).

**Table 3.** Comparison of adipogenic responses

Contraceptive chemical	Maximum responses (TG accumulation and preadipocyte proliferation) across adipogenesis models									
	3T3-L1 TG	3T3-L1 PP	Female 1 TG	Female 1 PP	Female 2 TG	Female 2 PP	Male 1 TG	Male 1 PP	Male 2 TG	Male 2 PP
Estrogens										
17α-Ethinyl-estradiol	0.4%	0.0%	34.2%*^a^*	40.8%	104.5%*^a^*	11.0%*^a^*	18.1%*^a^*	8.0%	96.1%*^a^*	23.4%*^a^*
Estetrol	2.5%	0.0%	4.6%	9.0%	32.9%*^a^*	7.3%*^a^*	8.6%*^a^*	23.3%*^a^*	5.3%*^a^*	14.2%*^a^*
Progestins										
Segesterone acetate (Nesterone)	145.5%*^a^*	53.1%*^a^*	7.8%	4.9%	4.1%	6.2%	7.1%	13.1%*^a^*	5.1%	1.3%
Etonogestrel	79.3%*^a^*	71.0%*^a^*	0.0%	9.0%	17.1%*^a^*	5.1%	12.8%*^a^*	0.0%	2.9%	5.4%
Drospirenone	29.1%	18.8%	9.2%*^a^*	6.5%	18.6%*^a^*	6.7%	19.6%*^a^*	21.0%*^a^*	21.3%*^a^*	7.1%
Ulipristal acetate	104.8%*^a^*	36.9%*^a^*	3.6%	2.2%	11.7%	3.4%	14.7%*^a^*	16.3%*^a^*	12.1%*^a^*	15.1%*^a^*
Levonorgestrel	91.4%*^a^*	15.5%	2.4%	0.0%	6.5%	8.1%	1.4%	19.0%*^a^*	52.5%*^a^*	7.9%*^a^*
Norelgestromin	44.3%*^a^*	53.7%*^a^*	8.4%	0.0%	13.6%*^a^*	3.0%	6.8%	12.4%*^a^*	8.6%	2.5%
Norgestimate	90.5%*^a^*	40.4%*^a^*	0.0%	7.2%	2.6%	0.0%	5.7%	2.8%	65.4%*^a^*	2.0%
Medroxyprogesterone acetate	137.2%*^a^*	83.9%*^a^*	27.7%*^a^*	5.4%	48.1%*^a^*	5.0%	5.5%	5.9%	14.7%*^a^*	14.3%*^a^*
Norethindrone	23.6%*^a^*	19.0%*^a^*	2.0%	2.4%	44.5%*^a^*	0.0%	3.2%	0.0%	10.5%*^a^*	14.6%*^a^*
Norethisterone acetate	1.0%	14.7%*^a^*	3.9%	0.0%	59.3%*^a^*	2.1%	7.7%	4.6%	18.2%*^a^*	20.4%*^a^*
Mixtures										
Etonogestrel + EE2	153.0%*^a^*	37.8%*^a^*	16.4%*^a^*	1.4%	15.1%*^a^*	16.0%*^a^*	7.9%	29.3%*^a^*	8.2%	5.9%
Norgestimate + EE2	116.9%*^a^*	2.4%	2.4%	0.0%	10.2%*^a^*	6.5%	5.8%	37.8%*^a^*	5.6%	3.4%
Norethisterone acetate + EE2	11.9%*^a^*	0.0%	2.6%	6.7%	25.8%*^a^*	14.9%*^a^*	4.5%	34.6%*^a^*	9.2%	5.6%
Nesterone + EE2	143.2%*^a^*	40.8%*^a^*	5.6%	14.9%*^a^*	17.5%*^a^*	13.9%*^a^*	2.2%	42.8%*^a^*	4.4%	9.0%
Norelgestromin + EE2	121.0%*^a^*	8.3%	5.0%	6.8%	16.8%*^a^*	15.5%*^a^*	3.2%	45.2%*^a^*	5.3%	16.0%*^a^*
Drospirenone + EE2	52.1%*^a^*	0.0%	6.6%	0.0%	10.0%	0.0%	3.3%	0.0%	10.4%*^a^*	14.5%*^a^*
Drospirenone + E4	86.6%*^a^*	0.0%	12.1%*^a^*	5.1%	6.3%	0.0%	7.9%*^a^*	22.0%*^a^*	13.6%*^a^*	19.2%*^a^*
Control chemicals										
17β-Estradiol	37.2%*^a^*	5.0%	1.9%	10.3%	52.1%*^a^*	15.4%*^a^*	1.8%	15.1%*^a^*	16.2%*^a^*	7.9%*^a^*
Progesterone	206.9%*^a^*	73.3%*^a^*	20.8%*^a^*	7.1%	26.8%*^a^*	8.1%*^a^*	23.4%*^a^*	3.1%	34.5%*^a^*	0.0%
17β-Estradiol + Progesterone	127.0%*^a^*	27.7%*^a^*	3.0%	5.2%	30.9%*^a^*	13.8%*^a^*	2.1%	15.7%	3.9%	12.8%*^a^*

Maximal efficacy in adipogenesis assays across models (eg, maximal % activity relative to controls for both triglyceride accumulation [TG] and preadipocyte proliferation [PP]) for the purposes of comparisons in responses between 3T3-L1 murine preadipocytes and 4 lots of human mesenchymal stem cells (additional details on mesenchymal stem cells available in Table 2).

Abbreviations: E4, estetrol; EE2, 17α-ethinyl-estradiol.

^*a*^Indicates significant increase over vehicle control response. *P* < .05, as per Kruskal–Wallis in GraphPad Prism 10.

**Figure 4.**
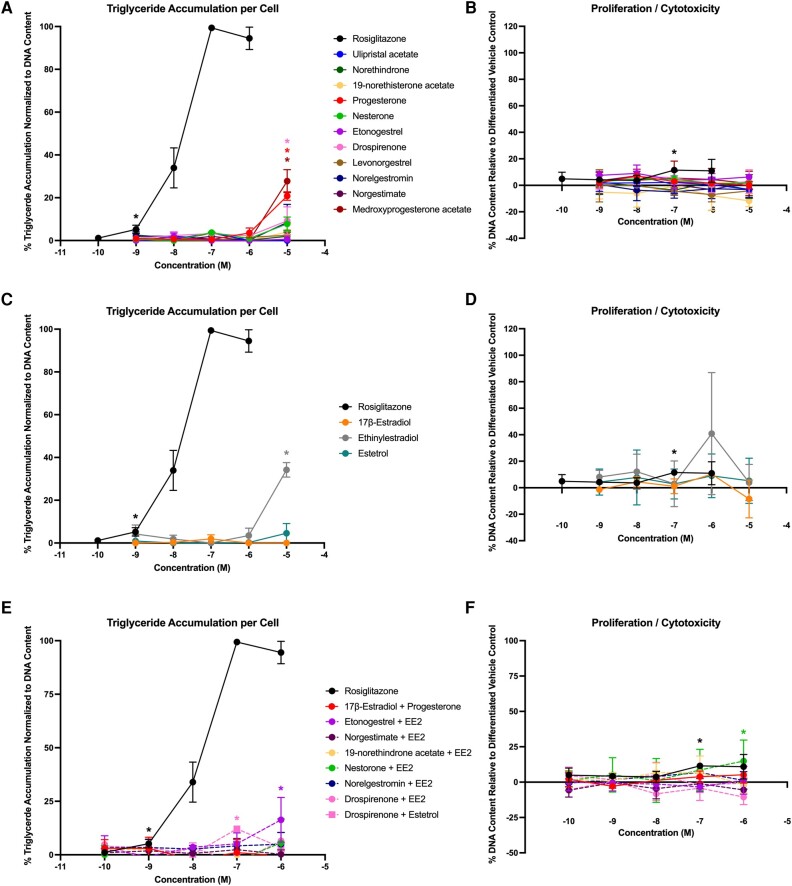
Adipogenic responses of female 1 human mesenchymal stem cells. Zenbio human mesenchymal stem cells (female, lot No. HBMMSC071819A) were differentiated as described in “Materials and Methods” and assessed for adipocyte differentiation (Nile Red staining of lipid accumulation) and cell proliferation (Hoechst staining) after 21 days of differentiation while exposed to various contraceptives or their mixtures at a range of concentrations. Percentage normalized triglyceride accumulation per well relative to maximal response for rosiglitazone (normalized to DNA content; A, C, E). Increase (cell proliferation) or decrease (potential cytotoxicity) in DNA content relative to vehicle control (B, D, F). Contraceptive progestins (A, B), estrogens (C, D), and combined contraceptive mixtures (E, F). Data are presented as mean ± SEM from 3 independent experiments. *Indicates lowest concentration with significant increase over vehicle control response; *P* less than .05, as per Kruskal-Wallis in GraphPad Prism 10.

**Figure 5.**
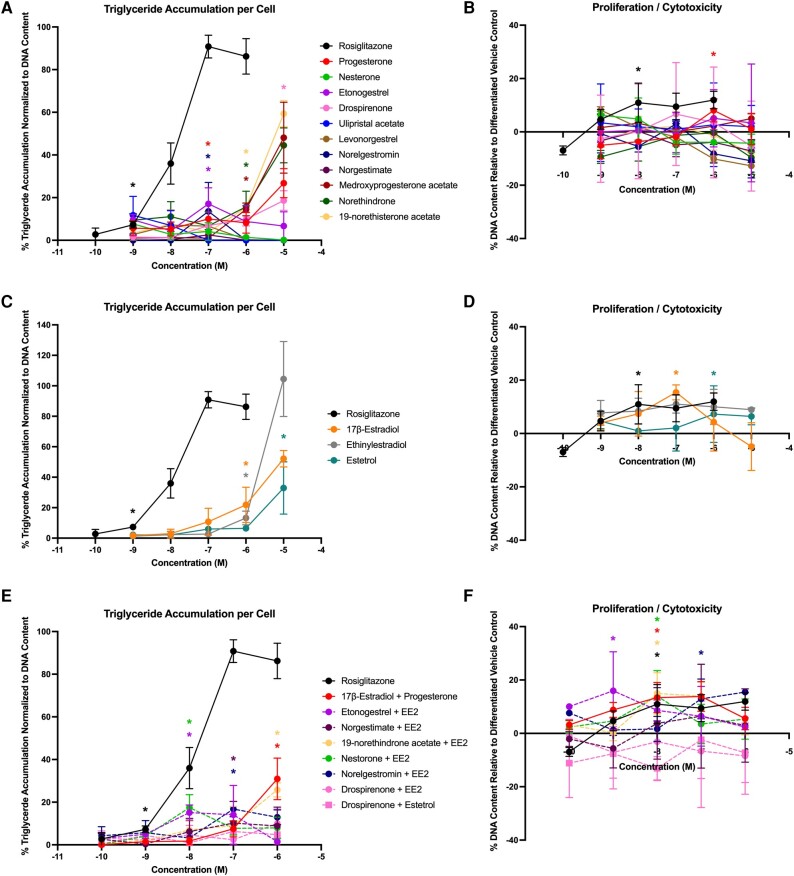
Adipogenic responses of female 2 human mesenchymal stem cells. ScienCell human mesenchymal stem cells (female, lot No. 31886) were differentiated as described in “Materials and Methods” and assessed for adipocyte differentiation (Nile Red staining of lipid accumulation) and cell proliferation (Hoechst staining) after 21 days of differentiation while exposed to various contraceptives or their mixtures at a range of concentrations. Percentage normalized triglyceride accumulation per well relative to maximal response for rosiglitazone (normalized to DNA content; A, C, E). Increase (cell proliferation) or decrease (potential cytotoxicity) in DNA content relative to vehicle control (B, D, F). Contraceptive progestins (A, B), estrogens (C, D), and combined contraceptive mixtures (E, F). Data are presented as mean ± SEM from 3 independent experiments. *Indicates lowest concentration with significant increase over vehicle control response; *P* less than .05, as per Kruskal-Wallis in GraphPad Prism 10.

To confirm adipogenic effects and support translation to human health determinations, each of the contraceptives and mixtures were tested in 4 different sourced human bone marrow–derived mesenchymal stem cell lines (see Tables 2 and 3). In Zenbio-sourced cells (female 1), 3 of the progestins promoted significant triglyceride accumulation (progesterone, medroxyprogesterone acetate, and drospirenone), with activity ranging from 20% to 28% (Fig. 4A); none of the progestins promoted preadipocyte proliferation (Fig. 4B). One of the estrogens, EE2, promoted significant triglyceride accumulation (34%; Fig. 4C), while none of them significantly promoted significant proliferation (Fig. 4D). Two of the mixtures induced significant triglyceride accumulation (drospirenone + EE2 and etonogestrel + EE2; Fig. 4E) and one promoted significant preadipocyte proliferation (nesterone + EE2; Fig. 4F). In ScienCell-sourced cells (female 2), 7 of the progestins promoted significant triglyceride accumulation (progesterone, etonogestrel, drospirenone, norelgestromin, medroxyprogesterone acetate, norethindrone, and norethisterone acetate), with activity ranging from 14% to 59% increased triglyceride accumulation (Fig. 5; see Table 3); only progesterone was able to stimulate significant proliferation. All 3 estrogens were active on triglyceride accumulation with 33% to 105% increased triglyceride accumulation and 7% to 15% increased proliferation. Among the mixtures, 6 of the 8 promoted significant increases in triglyceride accumulation (10%-31%) and 5 of the 8 promoted significant proliferation (14%-16%). The drospirenone + estrogen mixtures were inactive for both adipogenic measures in this cell model. While this mirrored relatively low activity for drospirenone alone, the estrogens were all moderate to high activity alone in this donor-sourced model.

**Figure 6.**
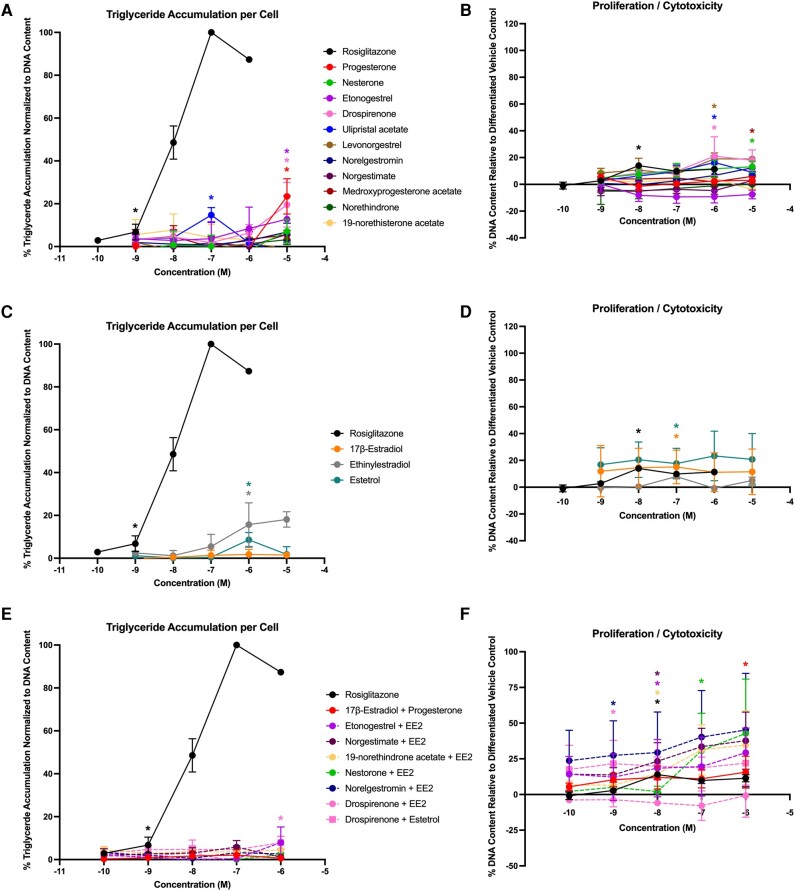
Adipogenic responses of male 1 human mesenchymal stem cells. Lonza human mesenchymal stem cells (male, lot No. 19TL155677) were differentiated as described in “Materials and Methods” and assessed for adipocyte differentiation (Nile Red staining of lipid accumulation) and cell proliferation (Hoechst staining) after 21 days of differentiation while exposed to various contraceptives or their mixtures at a range of concentrations. Percentage normalized triglyceride accumulation per well relative to maximal response for rosiglitazone (normalized to DNA content; A, C, E). Increase (cell proliferation) or decrease (potential cytotoxicity) in DNA content relative to vehicle control (B, D, F). Contraceptive progestins (A, B), estrogens (C, D), and combined contraceptive mixtures (E, F). Data are presented as mean ± SEM from 3 independent experiments. *Indicates lowest concentration with significant increase over vehicle control response; *P* less than .05, as per Kruskal-Wallis in GraphPad Prism 10.

**Figure 7.**
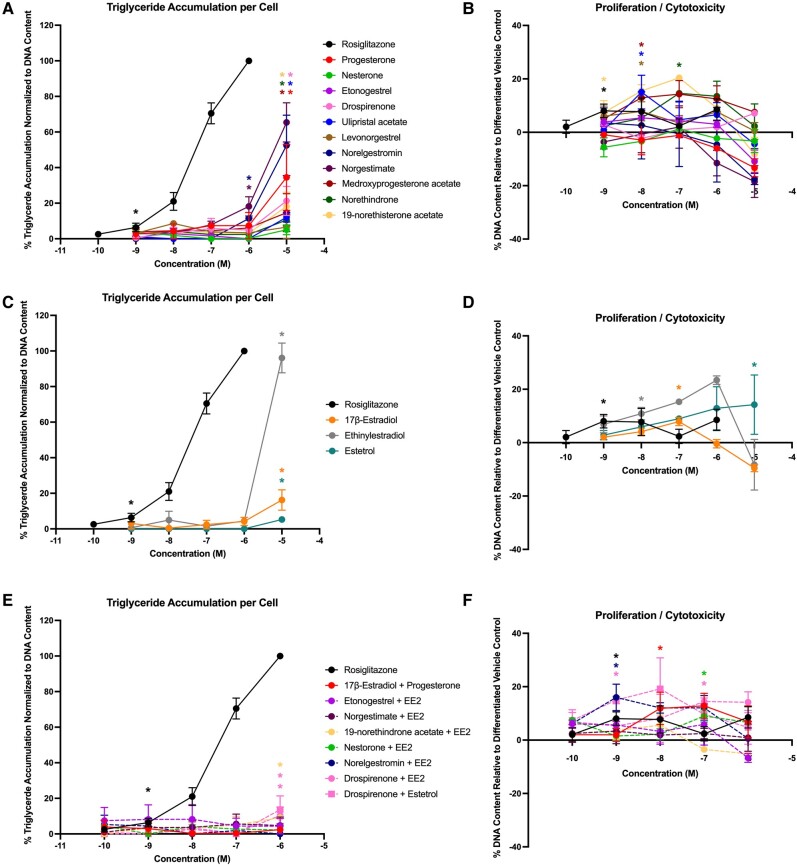
Adipogenic responses of male 2 human mesenchymal stem cells. ScienCell human mesenchymal stem cells (male, lot No. 30579) were differentiated as described in “Materials and Methods” and assessed for adipocyte differentiation (Nile Red staining of lipid accumulation) and cell proliferation (Hoechst staining) after 21 days of differentiation while exposed to various contraceptives or their mixtures at a range of concentrations. Percentage normalized triglyceride accumulation per well relative to maximal response for rosiglitazone (normalized to DNA content; A, C, E). Increase (cell proliferation) or decrease (potential cytotoxicity) in DNA content relative to vehicle control (B, D, F). Contraceptive progestins (A, B), estrogens (C, D), and combined contraceptive mixtures (E, F). Data are presented as mean ± SEM from 3 independent experiments. *Indicates lowest concentration with significant increase over vehicle control response; *P* less than .05, as per Kruskal-Wallis in GraphPad Prism 10.

In Lonza-sourced cells (male 1), 4 of the progestins promoted triglyceride accumulation (progesterone, drospirenone, etonogestrel, and ulipristal acetate), with activity ranging from 13% to 23% (Fig. 6A; see Table 3). Five of the progestins promoted significant preadipocyte proliferation, ranging from 13% to 21% (Fig. 6B). Two of the 3 estrogens promoted significant triglyceride accumulation (EE2 and E4; Fig. 6C) and 2 promoted significant preadipocyte proliferation (17β-E2 and E4; Fig. 6D). One of the mixtures (drospirenone + E4) promoted significant triglyceride accumulation (Fig. 6E) and 7 of the 8 mixtures induced significant preadipocyte proliferation (Fig. 6F). In ScienCell-sourced cells (male 2), 8 of the progestins promoted significant triglyceride accumulation (progesterone, drospirenone, ulipristal acetate, levonorgestrel, norgestimate, medroxyprogesterone acetate, norethindrone, and norethisterone acetate) with effects ranging from 11% to 65% (Fig. 7, Table 3), whereas 4 promoted significant proliferation (ulipristal acetate, medroxyprogesterone acetate, norethindrone, and norethisterone acetate) at rates from 8% to 20%. All 3 estrogens significantly promoted both triglyceride accumulation (5%-96%) and preadipocyte proliferation (8%-23%). Among the mixtures, only 2 demonstrated significant triglyceride accumulation (both drospirenone mixtures, 10%-14%) and 4 exhibited significant proliferation (estradiol + progesterone, both drospirenone mixtures, and norelgestromin + EE2; 13%-19%).

### Mechanistic Assessment of Adipogenic Effects

**Figure 8.**
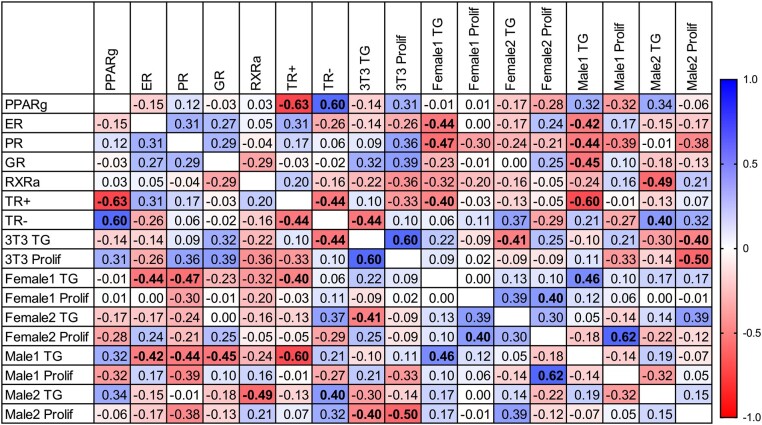
Correlations between bioactivities induced by contraceptive chemicals and mixtures. Spearman correlations were performed to assess associations between bioactivities resulting from contraceptive chemical exposures, either individually or in combined mixtures. Activation or inhibition of receptor bioactivities for the peroxisome proliferator activated receptor gamma (PPARg), estrogen receptor alpha (ER), progesterone receptor B (PR), glucocorticoid receptor (GR), retinoid X receptor (RXRa), and thyroid hormone receptor (TR; + = agonism, – = antagonism). 3T3 = 3T3-L1 murine preadipocytes. TG = triglyceride accumulation, prolif = proliferation (DNA content). Female 1/2 and Male 1/2 are human mesenchymal stem cells described in Table 2. Bold numbers represent *P* values less than .05. Colors represent the scale of the effects, with darker blue colors denoting more positive correlations and darker red denoting more negative correlations.

Spearman correlations were performed to assess the degree of interrelationship that existed among different bioassays (Fig. 8). Triglyceride accumulation had a significant negative association with ER activation in the female 1 and male 1 hMSC models, with weaker (nonsignificant) trends observed in the other 3 adipogenic models. This was mirrored with significant negative associations in PR agonism for these same 2 adipogenesis models, though this was less consistent across the other models, with a nonsignificant positive association in 3T3-L1 cells, a nonsignificant negative correlation in female 2 cells, and a lack of directionality of association in male 2 cells. GR activation had a significant negative association with triglyceride accumulation in male 1 cells; nonsignificant negative correlations were observed in female 1 and male 2 cells, and a nonsignificant positive correlation was observed in 3T3-L1 cells. A significant negative correlation was observed for RXR activation and triglyceride accumulation in male 2 cells, with nonsignificant negative correlations also noted in 3T3-L1, female 1, female 2, and male 1 cells. TR agonism and triglyceride accumulation were negatively correlated for female 1 and male 1 cells, whereas TR antagonism had a significant negative correlation with triglyceride accumulation for 3T3-L1 cells and a significant positive correlation in male 2 cells. Among reporter gene assay data, PPARγ activity was positively correlated with TR antagonism and negatively correlated with TR agonism. As expected, TR agonism and antagonism were negatively correlated. Among the adipogenic measures, 3T3-L1 triglyceride accumulation was positively correlated with 3T3-L1 proliferation and negatively correlated with female 2 and male 2 proliferation. 3T3-L1 proliferation was negatively correlated with male 2 proliferation. Female 1 triglyceride accumulation was positively correlated with male 1 triglyceride accumulation, and female 1 proliferation with female 2 proliferation. No other significant correlations were observed between adipogenic activities.

**Figure 9.**
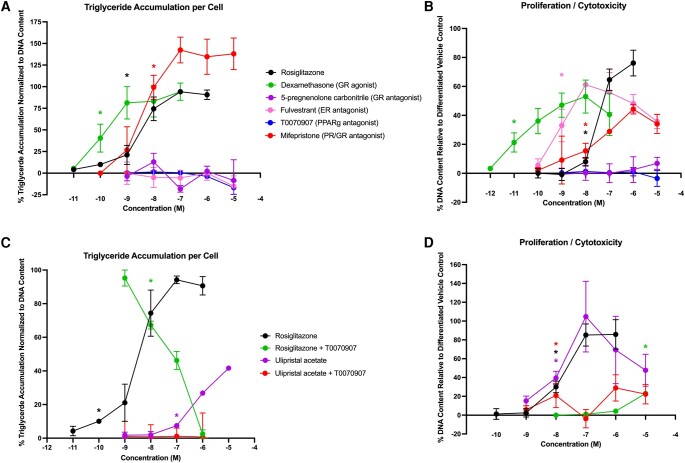
Adipogenic responses of control chemicals and PPARg antagonist coexposure “recovery” assessment. Zenbio 3T3-L1 cells were differentiated as described in “Materials and Methods” and assessed for adipocyte differentiation (Nile Red staining of lipid accumulation) and cell proliferation (Hoechst staining) after 10 days of differentiation while exposed to various control chemicals, contraceptives, or their mixtures at a range of concentrations. Percentage normalized triglyceride accumulation per well relative to maximal response for rosiglitazone (normalized to DNA content; A). Increase (cell proliferation) or decrease (potential cytotoxicity) in DNA content relative to vehicle control (B). Recovery assessment for peroxisome proliferator activated receptor gamma (PPARg) mechanistic assessment. Cells were coexposed to rosiglitazone and ulipristal acetate with and without coexposure of 10 μM T0070907 and assessed for adipogenic responses to determine the role of PPARg in the proadipogenic effects (C, D). Data are presented as mean ± SEM from 3 independent experiments. *Indicates lowest concentration with significant increase over vehicle control response; *P* less than .05, as per Kruskal-Wallis in GraphPad Prism 10 (A, B). Lowest concentration with significant change from contraceptive alone; *P* less than .05, following coexposure to 10 μM T0070907 (C, D).

Several coexposure experiments were performed to better assess potential mechanistic pathways underlying the observed proadipogenic effects. Control chemical responses were delineated in the 3T3-L1 cell model (Fig. 9A and 9B), with rosiglitazone, dexamethasone, and mifepristone inducing strong proadipogenic effects on triglyceride accumulation (see Fig. 9A). Fulvestrant (ER antagonist), T0070907 (PPARγ antagonist), and 5-pregnenolone carbonitrile (5PC; GR antagonist) had no significant effects on triglyceride accumulation. Significant effects on triglyceride accumulation were unsuspected for mifepristone, and prevented its use in recovery experiments; with a lack of an efficacious and specific PR antagonist, we opted to perform some experiments with 5-pregnenolone carbonitrile, which is a nonadipogenic GR-specific antagonist. Interestingly, while this same pattern of activity was apparent on the preadipocyte proliferation metric (Fig. 9B), fulvestrant additionally induced a robust increase in DNA content. T0070907 acted as expected to inhibit rosiglitazone-induced proadipogenic effects (Fig. 9C and 9D). T0070907 was also tested with the proadipogenic PPARγ-activating contraceptive, ulipristal acetate; T0070907 completely inhibited the effects of ulipristal acetate on triglyceride accumulation and significantly inhibited the effects on preadipocyte proliferation (see Fig. 9C and 9D). While this suggests a causal role for PPARγ, there is some concern that due to the central role of PPARγ in adipogenesis, this may not be a specific test for mechanism and could be a nonspecific inhibition of adipogenesis itself. As a result, further specificity evaluations focused on GR and ER, for which specific mechanism inhibition could be demonstrated more readily.

**Figure 10.**
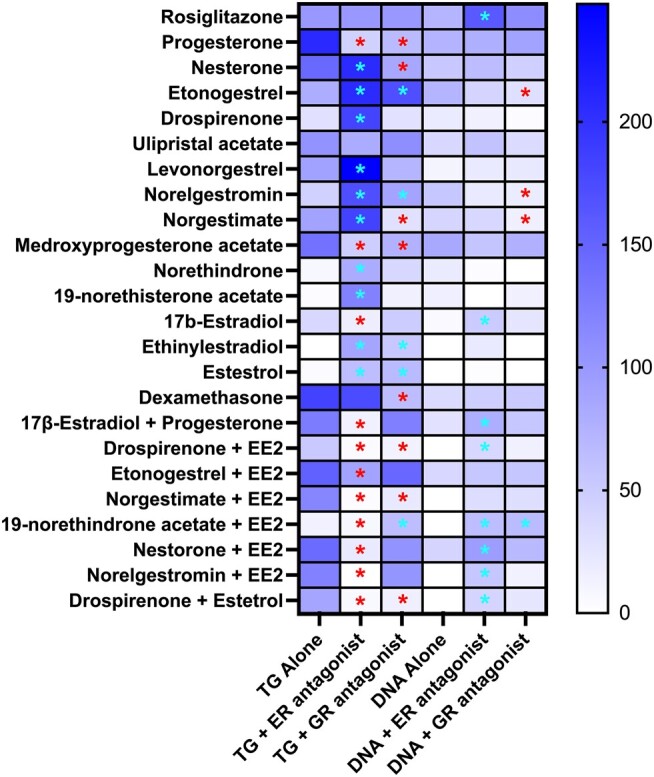
Mechanistic assessment of proadipogenic effects in 3T3-L1 cells. Zenbio 3T3-L1 cells were differentiated as described in “Materials and Methods” and assessed for adipocyte differentiation (Nile Red staining of lipid accumulation) and cell proliferation (Hoechst staining) after 10 days of differentiation while exposed to various contraceptives or their mixtures at a range of concentrations with c-exposures to 10 μM fulvestrant (estrogen receptor antagonist) or 10 μM 5-pregnenolone carbonitrile (glucocorticoid receptor antagonist). Percentage normalized triglyceride accumulation per well relative to maximal response for rosiglitazone (normalized to DNA content, maximal efficacy). Increase (cell proliferation) in DNA content relative to vehicle control (maximal increase). Data are presented as mean ± SEM from 3 independent experiments. *Indicates significant change with coexposure relative to the contraceptive or mixture by itself; *P* less than .05, as per Kruskal-Wallis in GraphPad Prism 10. Red denotes significant decrease relative to individual contraceptive and blue denotes significant increase. Columns listing “+ ER antagonist” were coexposed with fulvestrant and columns listing “+ GR antagonist” were coexposed with 5-pregnenolone carbonitrile as described earlier for each.

**Figure 11.**
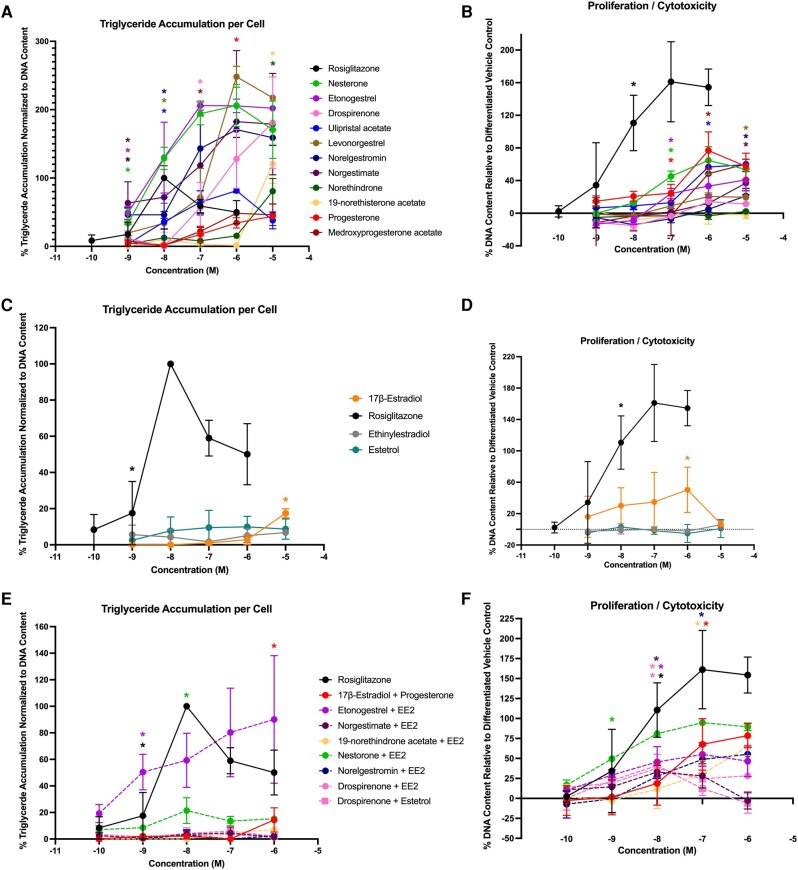
Estrogen receptor mediated proadipogenic activity of contraceptives through fulvestrant coexposures. Zenbio 3T3-L1 cells were differentiated as described in “Materials and Methods” and assessed for adipocyte differentiation (Nile Red staining of lipid accumulation) and cell proliferation (Hoechst staining) after 10 days of differentiation while exposed to various contraceptives or their mixtures at a range of concentrations while coexposed to 10 μM fulvestrant. Percentage normalized triglyceride accumulation per well relative to maximal response for rosiglitazone (normalized to DNA content; A, C, E). Increase (cell proliferation) or decrease (potential cytotoxicity) in DNA content relative to vehicle control (B, D, F). Responses provided were the progestins (A, B), estrogens (C, D), and combined mixtures (E, F). Data are presented as mean ± SEM from 3 independent experiments. *Indicates lowest concentration with significant change from contraceptive alone; *P* less than .05, as per Kruskal-Wallis in GraphPad Prism 10. All treatments depicted in this figure were coexposed with 10 μM fulvestrant. These chemicals without fulvestrant cotreatment (same cell line) are provided in Fig. 3.

**Figure 12.**
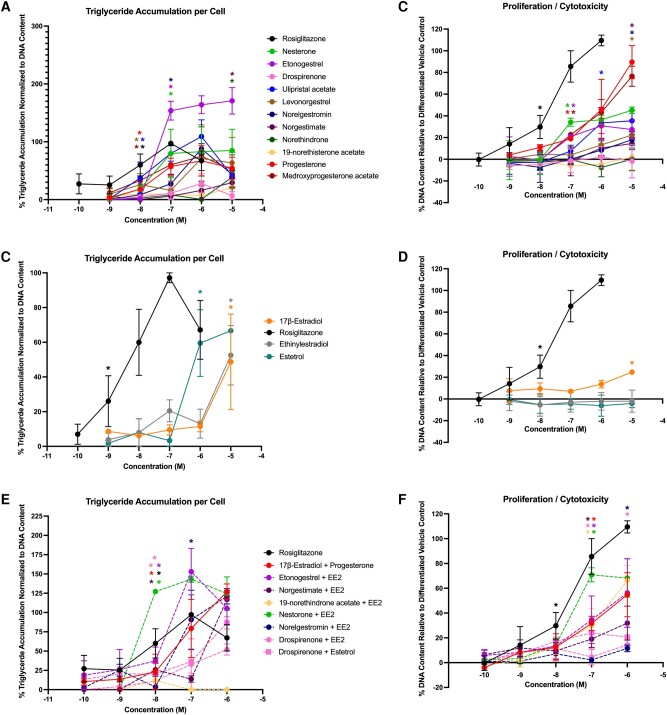
Glucocorticoid receptor mediated proadipogenic activity of contraceptives through 5-pregnenolone carbonitrile coexposures. Zenbio 3T3-L1 cells were differentiated as described in Methods and assessed for adipocyte differentiation (Nile Red staining of lipid accumulation) and cell proliferation (Hoechst staining) after 10 days of differentiation while exposed to various contraceptives or their mixtures at a range of concentrations while coexposed to 10 μM 5-pregnenolone carbonitrile. Percentage normalized triglyceride accumulation per well relative to maximal response for rosiglitazone (normalized to DNA content; A, C, E). Increase (cell proliferation) or decrease (potential cytotoxicity) in DNA content relative to vehicle control (B, D, F). Responses provided were the progestins (A, B), estrogens (C, D), and combined mixtures (E, F). Data are presented as mean ± SEM from 3 independent experiments. *Indicates lowest concentration with significant change from contraceptive alone; *P* less than .05, as per Kruskal-Wallis in GraphPad Prism 10. All treatments depicted in this figure were coexposed with 10 μM 5-pregnenolone carbonitrile. Chemicals without 5-pregnenolone carbonitrile cotreatment (same cell line) are provided in Fig. 3.

Each individual contraceptive and mixture were coexposed to 10 μM fulvestrant across their full-dose response range (Figs. 10 and 11). Cotreatment with fulvestrant significantly inhibited the triglyceride accumulation induced by E2, supporting the estrogenic proadipogenic mechanism. Fulvestrant cotreatment also significantly inhibited the triglyceride accumulation induced by every contraceptive mixture. More surprisingly, the effects of fulvestrant on the progestins were mixed. Fulvestrant inhibited the proadipogenic effects of progesterone (79% reduction) and medroxyprogesterone acetate (65% reduction), had no effect on ulipristral acetate, and exacerbated the triglyceride accumulation induced by the 8 other progestins (nesterone, etonogestrel, drospirenone, levonorgestrel, norelgestromin, norgestimate, norethindrone, and 19-norethisterone acetate). Interestingly, the inhibitory effects of fulvestrant on 3T3-L1 triglyceride accumulation were not mirrored for preadipocyte proliferation. Fulvestrant increased the DNA content for rosiglitazone, estradiol, and for 6 of the contraceptive mixtures. Every contraceptive and each mixture were then additionally coexposed to 10 μM 5PC across their full-dose response range (see Fig. 10, Fig. 12). 5PC significantly inhibited the triglyceride accumulation induced by dexamethasone. Interestingly, 5PC significantly increased the triglyceride accumulation induced by EE2 and E4, without any significant effect on E2. Mixed effects were observed on the progestins, with 5PC inhibiting triglyceride accumulation induced by 4 progestins (progesterone, nesterone, norgestimate, and medroxyprogesterone acetate), but increasing 2 others (etonogestrel and norelgestromin). 5PC inhibited triglyceride accumulation induced by 3 mixtures and increased 1, demonstrating mixed effects (though mostly inhibitory on progestins). Effects on preadipocyte proliferation were less marked, with significant reductions in 3 progestin-mediated DNA increases and a significant increase for the 19-norethindrone acetate + EE2 mixture.

## Discussion

The use of hormonal contraceptives has become prevalent worldwide as an effective and convenient method of family planning as well as for therapeutic use for a number of medical issues. Despite their widespread use, recent concerns have arisen regarding their potential effect on metabolic health, particularly weight gain, which is a commonly reported reason for discontinuation of these contraceptives. Epidemiological studies have yielded mixed results, and there has been limited research on the effects of hormonal contraceptives on metabolic health, especially when taken at low concentrations over extended periods. This study directly assessed the adipogenic activity of common hormonal contraceptive chemicals and their mixtures and investigated their potential mechanisms of action in an effort to help elucidate potential effects on metabolic health.

First and foremost, every current-use hormonal contraceptive and every mixture was active on adipogenic outcomes, as demonstrated by triglyceride accumulation and/or preadipocyte proliferation. Progestins such as nesterone, etonogestrel acetate, and medroxyprogesterone acetate induced significant and robust effects both on triglyceride accumulation and preadipocyte proliferation in the 3T3-L1 model, with the others demonstrating effects only on one metric or more moderate effects overall. Drospirenone and 19-norethisterone acetate exhibited no significant activity on triglyceride accumulation, whereas all 11 progestins exhibited significant effects on preadipocyte proliferation. These results were largely confirmed using hMSCs from 4 separate donors. First and foremost, limited significant correlations were observed across responses in the 4 hMSC models, suggesting that there may be differences via sex, race, age, and/or other factors that influence adipogenic activities in human models. In terms of consistency, drospirenone was active for triglyceride accumulation across all 5 models and active for proliferation in only 2. Medroxyprogesterone acetate was active for triglyceride accumulation in 4 of 5 models and proliferation in 2 of 5. EE2 was highly active for triglyceride accumulation in the 4 hMSC models and for proliferation in 2 of these, whereas it was inactive in 3T3-L1 cells. E4 was similarly active in 3 of 4 hMSC models both for triglyceride accumulation and proliferation. Progesterone was active for triglyceride accumulation in all 5 models and on proliferation in 3 and E2 was active for each metric in 3 of the 5. Several chemicals and/or mixtures may have some effects via age or sex, with the female/male 2 donors being active for norethindrone and norethisterone acetate, but inactive in the female hMSC lines. Age could play a role in the differences observed for ulipristal acetate, levonorgestrel (both males active, females inactive), and the etonogestrel + EE2 mixture (females active, males inactive). Further research is needed to better examine differences among the models that may result in the variance observed here in the responses. It is important to note that every chemical or mixture was active in one or more human MSC models, substantiating the 3T3-L1 results but shedding light on inherent variability that may exist between human donor cell lines that requires further investigation in future studies.

The causal mechanism(s) underlying the proadipogenic effects of the progestins was not completely clear. It was not possible to examine the relative role of PR-mediated agonism, due to lack of specific control antagonists that did not also promote adipogenesis, though we did find mixed effects for ER and GR antagonism. Cotreatment with fulvestrant (ICI 182 780), an ER antagonist, appeared to enhance the triglyceride accumulation induced by 8 of the 11 progestins (2 were significantly inhibited and 1 had no significant change). Of those that were not enhanced, ulipristal acetate was seemingly mediated by PPARγ activation, which we demonstrated with T0070907 cotreatment experiments. The 2 that were inhibited, medroxyprogesterone acetate and progesterone (79% and 65% inhibition of triglyceride accumulation, respectively), had mixed bioactivities. Progesterone had no apparent effects on any of the other examined receptor bioactivities, suggesting that this may work exclusively through PR signaling—though the coexposure requires further investigation to elucidate. Medroxyprogesterone acetate was not active on ER, but was very active on GR and as a TRβ antagonist. This would explain why medroxyprogesterone was significantly inhibited by 5PC coexposure, but does not explain why it was affected by fulvestrant. There are some complex dynamics between ER and PR, and some estrogens and antiestrogens can increase PR expression (55), which might subsequently influence adipogenic activities. There is also an intimate relationship between GR and PR, with both receptors recognizing the same 15-base pair DNA sequence and steroid-specific gene activation seemingly regulated primarily by differential expression of receptors (56). There has been previous research demonstrating that the effects of progesterone and medroxyprogesterone acetate occurred through GR and not PR, which aligns with our data demonstrating a difference for these 2 chemicals in the 5PC cotreatment effects (57). Perhaps most notably, however, was the diversity of receptor bioactivities for the progestins; 8 activated ERα, 5 activated GR, 1 activated PPARγ, and 7 antagonized TRβ. This likely contributes to the mixed responses for epidemiological assessments, as these chemicals do not act similarly across diverse nuclear receptors.

Three estrogens were examined and they each demonstrated proadipogenic effects in one or more models. In 3T3-L1 cells, E2 promoted moderate effects on triglyceride accumulation and no effects on preadipocyte proliferation. In hMSCs, EE2 exhibited effects on triglyceride accumulation in Zenbio-sourced hMSCs and both EE2 and E4 promoted effects on triglyceride accumulation in Lonza-sourced hMSCs. Both E2 and E4 promoted significant preadipocyte proliferation only in the Lonza-sourced hMSCs, without any effects on preadipocyte proliferation in either of the other cell models. The differences in molecular pathways between 3T3-L1 cells and hMSCs are not well understood, so it is hard to critically evaluate why the disparities in activation may occur. The hMSCs were sourced from male and female donors, and there are well-described sex-specific effects on adipogenesis (25, 58-60), so that may explain some of the differences reported between these two cell lots. Overall, the adipogenic effects of the contraceptive estrogens were less robust than the contraceptive progestins, suggesting that these chemicals may play an outsized role in the reported metabolic health effects. This seems to be supported by the epidemiological literature, with the few studies generally finding positive outcomes for some or all of the progestins, separately from other hormonal contraceptives (29, 34, 36, 38).

The mechanistic assessment of adipogenic effects for estrogens revealed some intriguing results. Coexposure experiments with fulvestrant, an ER antagonist, indicated that ERα activation plays a significant role in the proadipogenic effects of estrogen-containing contraceptives and their mixtures. Fulvestrant significantly inhibited triglyceride accumulation induced by E2 and every single one of the contraceptive mixtures, supporting the hypothesis that estrogenic activity contributes to the proadipogenic effects of some estrogens and all of the combined hormonal contraceptives. Interestingly, cotreatment with 5PC seemed to enhance the proadipogenic effects of EE2 and E4, which was unexpected, whereas it did not significantly affect E2 (which was shown to be effectively inhibited by fulvestrant), suggesting some differences in mechanism of action between the endogenous E2 and the contraceptive estrogens, though surprisingly, E2 was the only estrogen that purportedly exhibited agonism for GR, which suggests this is likely to be nonspecific if the GR antagonist failed to inhibit the proadipogenic response. EE2 did demonstrate some antagonism for TRβ, which may explain some of the effects for this chemical, as well as PR activation (which E2 also exhibited). E4, other than agonism for ER, did not demonstrate any other significant bioactivities. This suggests that, given E4 did not have measurable effects on triglyceride accumulation in 3T3-L1 cells, this is likely mediated by ER. However, the mixture effects of E4 + 5PC causing enhanced triglyceride accumulation is unexpected and the mechanism requires further research to elucidate.

For the mixtures, 7 of the 8 mixtures induced significant triglyceride accumulation in 3T3-L1 cells and 3 of the 8 induced significant effects on preadipocyte proliferation (19-norethindrone acetate being the one inactive mixture). Mixtures were generally less active on triglyceride accumulation in hMSCs, with one mixture inducing triglyceride accumulation in Zenbio-sourced cells and one in Lonza-sourced cells (the same mixture, drospirenone + E4, a more novel contraceptive formulation). Preadipocyte proliferation in hMSCs was also varied; in Zenbio-sourced cells, only a single mixture promoted a significant increase in DNA content (nesterone + EE2), as compared with Lonza-sourced cells, where 7 of the 8 mixtures induced significant proliferative response. It is also helpful to examine whether the combinations exhibited greater responses than would be expected based on single component responses. Generally, these were not markedly dissimilar from single components, though 4 mixtures (drospirenone + EE2, drospirenone + E4, norelgestromin + EE2, and etonogestrel + EE2) were each significantly elevated relative to the 2 individual component contraceptives. These combinations should be explored further to better understand whether these responses are simply additive or potentially synergistic.

Interestingly, fulvestrant significantly inhibited the triglyceride accumulation induced by every single contraceptive mixture. This would suggest that the estrogenic component was the more significant contributor to the overall mixture activity, but EE2 was inactive in 3T3-L1 cells, so either there are unexpected mixture effects for EE2 when combined with progestins, or the fulvestrant was inhibiting the progestin component preferentially. However, independently, fulvestrant was able to inhibit only 2 progestins, while it actually acted to enhance the triglyceride accumulation of 8 other progestins. This apparent discrepancy requires further investigation to further elucidate. The mixtures appeared to exhibit greater potencies in combination than any of the component progestins or estrogens for ERα agonism, which supports a role of ER signaling in the mixture-induced proadipogenic effects. Further research should better delineate how ERα specifically acts to support the observed effects of the various contraceptives and their mixtures on the proadipogenic measures, via some combination of small interfering RNA knockdown, ERα-negative cell model testing, or other techniques. There were also some inhibitory effects for 5PC. Some of these, such as norgestimate and norgestimate + EE2, were both inhibited by 5PC cotreatment. Others that were inhibited in the mixture were not affected individually, suggesting that the mixtures may have unexpected effects or may activate different pathways than the single-component contraceptives when in combination.

Overall, this study highlights the multifaceted nature of the metabolic effects of hormonal contraceptives. The findings suggest that the composition of contraceptive mixtures, as well as the receptor activation and antagonism profiles, can have a significant effect on metabolic health outcomes in vitro. We report that hormonal contraceptives are not a homogenous group, and their effects vary depending on the specific compounds they contain. These results emphasize the need for further research to better understand the long-term metabolic implications of hormonal contraceptive use on reproductive-aged women, and a critical evaluation of potential metabolic health (or other health) effects in offspring from exposures during gestation. The study has several strengths, including the use of multiple models to assess adipogenesis and the examination of various hormonal contraceptives and their mixtures, particularly with mechanistic coexposure experiments to better delineate causal mechanisms. Very limited research has used these chemicals in adipogenic activity testing previously. More commonly, other model estrogens have been described as proadipogenic, such as diethylstilbestrol and E2 (15, 61). Progesterone and progesterone antagonists have also previously been described as proadipogenic in a variety of adipocyte models (62-65), which may be due to direct effects on adipocyte determination and differentiation 1/sterol regulatory element-binding protein 1c (66) and/or through direct activation of the glucocorticoid receptor (as has been described for progesterone and the PR antagonist, mifepristone (67)).

However, some limitations should be considered. The study primarily focused on in vitro assessments, and the translation of these findings to in vivo scenarios (and human health directly) requires further investigation. There are also some disparate responses on causal or contributory mechanisms that require further follow-up. Importantly, selective receptor modulation has been well described for many of the nuclear receptors examined here (68-72), with implications on our testing; these pharmaceuticals have diverse nuclear receptor bioactivity in our human cell lines, though these represent specific source tissues in the body and are thus susceptible to tissue microenvironment differences that may result in different ligand-induced responses than may be observed in other tissue types. As such, while we report a snapshot of agonism and/or antagonism for these pharmaceuticals, they may act entirely different in other cell lines. A classic example of this is that of tamoxifen, which acts as an ER agonist in bone and endometrium, yet acts as an antiestrogen in the breast. Human cell lines have also not been well characterized as adipogenesis models as of yet. There is diverse literature reporting interindividual differences in responses based on sex (25, 73-77), ethnicity (73, 78-81), physiology (eg, diabetes, obesity) (79, 81), age (82-85), adipose depot location (eg, subcutaneous vs visceral, thigh vs breast) (78, 86, 87), as well as other demographic variables, but these have never been evaluated systematically for differential responsiveness to environmental contaminants acting through specific molecular pathways for either human preadipocytes or hMSCs, as we have discussed previously (44).

In conclusion, this research provides valuable insights into the complex interactions between hormonal contraceptives and metabolic health outcomes. The results suggest that both progestins and estrogens within these contraceptives can influence adipogenesis, and the specific effects may vary based on the receptor activation profiles. Further research is warranted to elucidate the long-term consequences of hormonal contraceptive use on metabolic health and to explore the potential implications for children exposed to these contraceptives during gestation. Understanding these mechanisms is crucial for developing strategies to mitigate any adverse metabolic effects associated with hormonal contraceptive use and to promote reproductive health and well-being.

## Data Availability

Some or all data sets generated during and/or analyzed during the current study are not publicly available but are available from the corresponding author on reasonable request.

## Abbreviations

5PC: 5-pregnenolone carbonitrile
DMSO: dimethyl sulfoxide
E2: estradiol
E4: estetrol
EE2: ethinylestradiol
ERα: estrogen receptor alpha
GR: glucocorticoid receptor
hMSCs: human mesenchymal stem cells
IUD: intrauterine device
PPARγ: peroxisome proliferator activated receptor gamma
PR B: progesterone receptor B
RXRα: retinoid X receptor alpha
TRβ: thyroid hormone receptor beta
